# Immunotherapy using algal‐produced Ara h 1 core domain suppresses peanut allergy in mice

**DOI:** 10.1111/pbi.12515

**Published:** 2016-01-23

**Authors:** James A. Gregory, Ariel Shepley‐McTaggart, Michelle Umpierrez, Barry K. Hurlburt, Soheila J. Maleki, Hugh A. Sampson, Stephen P. Mayfield, M. Cecilia Berin

**Affiliations:** ^1^ Department of PediatricsIcahn School of Medicine at Mount SinaiNew YorkNYUSA; ^2^ Immunology InstituteIcahn School of Medicine at Mount SinaiNew YorkNYUSA; ^3^ U.S. Department of Agriculture, Agricultural Research ServiceSouthern Regional Research CenterNew OrleansLAUSA; ^4^ Mindich Child Health and Development InstituteIcahn School of Medicine at Mount SinaiNew YorkNYUSA; ^5^ Department of BiologyUniversity of California San DiegoLa JollaCAUSA; ^6^ Tisch Cancer InstituteIcahn School of Medicine at Mount SinaiNew YorkNYUSA

**Keywords:** algae, peanut, allergy, immunotherapy, biotechnology, recombinant protein, *Chlamydomonas reinhardtii*

## Abstract

Peanut allergy is an IgE‐mediated adverse reaction to a subset of proteins found in peanuts. Immunotherapy aims to desensitize allergic patients through repeated and escalating exposures for several months to years using extracts or flours. The complex mix of proteins and variability between preparations complicates immunotherapy studies. Moreover, peanut immunotherapy is associated with frequent negative side effects and patients are often at risk of allergic reactions once immunotherapy is discontinued. Allergen‐specific approaches using recombinant proteins are an attractive alternative because they allow more precise dosing and the opportunity to engineer proteins with improved safety profiles. We tested whether Ara h 1 and Ara h 2, two major peanut allergens, could be produced using chloroplast of the unicellular eukaryotic alga, *Chlamydomonas reinhardtii*. *C. reinhardtii* is novel host for producing allergens that is genetically tractable, inexpensive and easy to grow, and is able to produce more complex proteins than bacterial hosts. Compared to the native proteins, algal‐produced Ara h 1 core domain and Ara h 2 have a reduced affinity for IgE from peanut‐allergic patients. We further found that immunotherapy using algal‐produced Ara h 1 core domain confers protection from peanut‐induced anaphylaxis in a murine model of peanut allergy.

## Introduction

There is an urgent need to develop therapeutic approaches for treating food allergy, a disease for which there is no cure and the current standard of care is avoidance. A small number of foods, including milk, egg, wheat, shellfish, tree nut and peanut, account for the majority of food allergies (Soares‐Weiser *et al*., 2014), but accidental exposure is nonetheless difficult to avoid, especially for individuals with multiple allergies. Approximately 6–8% of children are affected, which is a significant increase in prevalence from recent decades (Panel *et al*., 2010). Fortunately, many food allergies are outgrown during childhood. Peanut allergy, however, is usually a lifelong condition that affects 1–2% of the population, has no diagnostic test to predict severity, and is the most common cause of fatal or near‐fatal anaphylaxis. Thus, the standard of care is strict avoidance and ready access to epinephrine.

Peanut allergy is an IgE‐mediated adverse reaction to specific proteins found in the legume *Arachis hypogaea* (Berin and Sampson, 2013). Thus far, sixteen proteins in *A. hypogaea* have been identified as allergens (Ara h 1–Ara h 17, Ara h4 was renamed to Ara h3.02; www.allergen.org); Ara h 1 and Ara h 2 are the dominant and best‐characterized peanut allergens to date. Peanut‐allergic patients exhibit a T_H_2‐polarized response to peanut and IgE that recognize one or more allergens (Flinterman *et al*., 2008, 2010). Upon exposure to peanut, IgE on tissue resident mast cells and circulating basophils cross‐link their cognate allergen causing rapid degranulation and release of histamine and inflammatory molecules (MacGlashan, 2008). This cascade leads to allergic reactions ranging from mild rash and gastrointestinal distress to fatal systemic anaphylaxis and organ failure.

Treatment of food allergies using allergen‐specific immunotherapy delivered by oral, sublingual or epicutaneous routes is a promising treatment option for food allergy (Wang and Sampson, 2012). Although the mechanism of protection remains unclear, immunotherapy aims to increase the threshold of allergen required to elicit an allergic response through repeated and escalating doses of the offending allergen in extract, powder or other form. The risk of adverse therapy‐induced side effects, including anaphylaxis, is high for peanut allergy. Crude peanut extracts complicate therapeutic approaches because they contain complex and variable mixtures of proteins. There may be components of peanut extracts that contribute to sensitization (Tordesillas *et al*., 2014). Immunotherapy using crude extracts can also increase the risk of future adverse reactions by introducing new IgE specificities to an allergic patient's IgE repertoire (Vickery *et al*., 2013) and, once patients discontinue therapy, adverse reactions return. Thus, peanut immunotherapy is not recommended in clinical practice (Sampson, 2013).

Recombinant proteins are an attractive alternative to native allergens for immunotherapy and allergy diagnostics (Codreanu *et al*., 2011). Recombinant allergens can be purified without concern for contamination by cross‐reactive peanut proteins. This facilitates accurate component testing of allergic patient IgE repertoires and more defined doses of individual allergens for immunotherapy. They also provide greater flexibility and specificity because the native polypeptide sequence serves only as a starting point. For example, recombinant allergens can be engineered to reduce IgE cross‐linking on mast cells and basophils that initiate allergic responses. Mutagenesis of critical amino acids in IgE binding epitopes of Ara h 1, Ara h 2 and Ara h3 reduced the binding to IgE from peanut‐allergic patients (Li *et al*., 2003a). Immunotherapy using rectally administered heat‐killed *Escherichia coli* that produce modified Ara h 1–3 mitigated peanut‐induced anaphylaxis in a murine peanut allergy model, possibly due to the adjuvant effect of using *E. coli* as a delivery vehicle. Similar results were observed after subcutaneous administration of modified Ara h 1–3 in *Listeria monocytogenes* (Li *et al*., 2003b). Indeed, protection from peanut allergy is associated with a shift from T_H_2 to T_H_1 immune responses to peanut. Unfortunately, phase I clinical trials demonstrated that allergens with modified IgE binding sites could still trigger reactions in humans when rectally administered using heat‐killed *E. coli* (Wood *et al*., 2013).

Recent advances in molecular biology and genetic engineering have expanded the breadth of organisms that can be used to produce recombinant proteins, some of which have characteristics that may be beneficial for food allergy immunotherapy. For example, *Lactococcus lactis*, a genetically tractable member of the lactic acid producing bacteria, is safe for oral delivery and is being studied for use as a probiotic. Ara h 2 produced and orally delivered to mice using *L. lactis* cells, which can promote an immunomodulatory effect to recombinant proteins (Neutra and Kozlowski, 2006), resulted in reduced peanut‐specific IgE production and T_H_2 cytokines when used prophylactically (Ren *et al*., 2014). Plants are also attractive platforms for producing allergens, both because many allergens are of plant origin and because they provide an inexpensive scalable system that is not prone to infection by human pathogens (Schmidt *et al*., 2008). *Nicotiana benthamiana*, a well‐established tobacco system, has been used to make allergens from birch pollen (Krebitz *et al*., 2000), mugwort pollen (Siegert *et al*., 2012) and apple (Krebitz *et al*., 2003). Hypoallergenic birch pollen allergens were recently made in rice (Ogo *et al*., 2014). Oral delivery of transgenic rice seeds containing T‐cell epitopes from Cry j 1 and Cry j 2, the major Japanese cedar pollen allergens, lowers vaccination‐induced IgE and T‐cell responses (Takagi *et al*., 2005).

The purpose of this study was to assess the feasibility of producing recombinant peanut allergens using the chloroplast of the unicellular eukaryotic green alga, *Chlamydomonas reinhardtii*. Relative to terrestrial plants, *C. reinhardtii* can be rapidly transformed into stable transgenic strains and scaled to large volumes using minimal growth media in fully contained photobioreactors. Thus, algal‐derived recombinant proteins could be produced quickly and inexpensively. Costs will be further reduced by advances in cultivation and harvesting lead by industrial algal production for biofuel and commercial products. The tools to express transgenes from the nuclear and chloroplast genomes, both of which have been fully sequenced, are readily available. Thus far, algae have been used to produce single chain antibodies (Mayfield *et al*., 2003), full‐length human antibodies (Tran *et al*., 2009), vaccine antigens (Surzycki *et al*., 2009), human therapeutic proteins (Rasala *et al*., 2010) and even multiprotein biosynthetic pathways (Noor‐Mohammadi *et al*., 2012). Here we demonstrate that *C. reinhardtii* can produce Ara h 1 and Ara h 2, two structurally distinct peanut allergens, and these recombinant allergens have reduced IgE binding compared to the native proteins. We further demonstrate that immunotherapy using algal‐produced Ara h 1 reduces anaphylaxis in a murine model of peanut allergy.

## Results

### Construction of transgenic chloroplasts in *Chlamydomonas reinhardtii*

**Figure 1:**
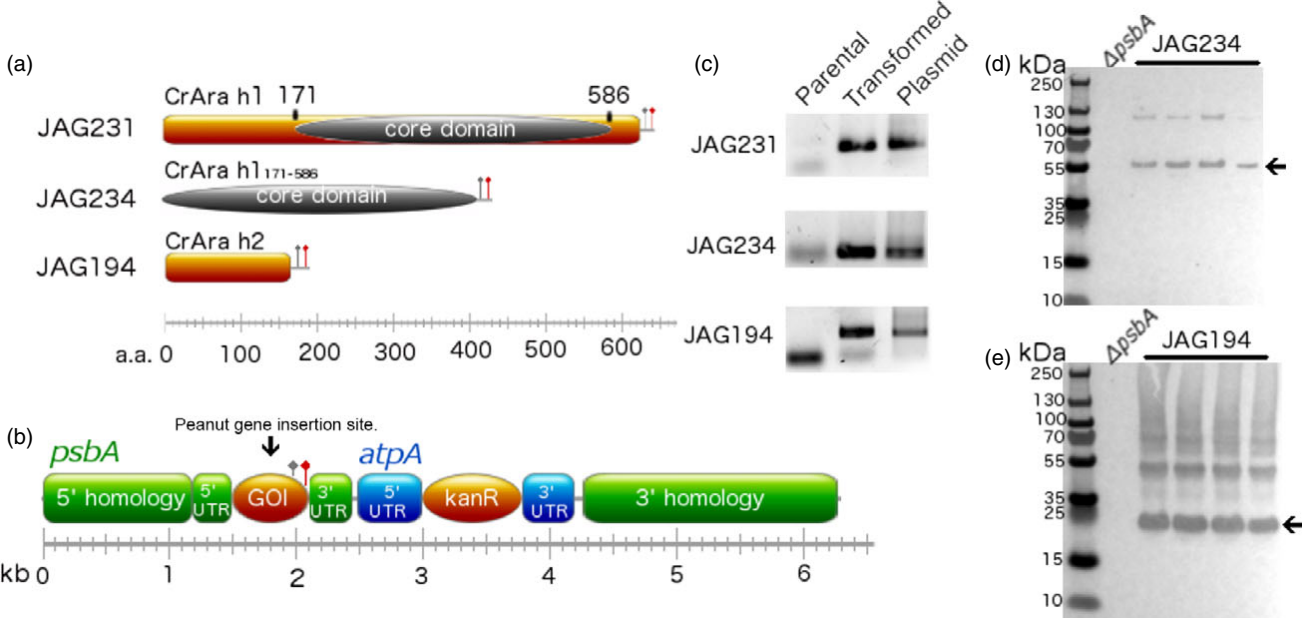
Construction and validation of transplastomic *Chlamydomonas reinhardtii* strains expressing *CrAra h 1* or *CrAra h 2*. (a) Diagram of recombinant Ara h 1, the core domain of Ara h 1, Ara h 2 and the (b) chloroplast transformation vector used to insert peanut allergen genes into the *psbA* locus of the *C. reinhardtii* plastid genome. Grey flag—TEV protease site. Red flag—FLAG affinity epitope. (c) Parental, transplastomic algal strains, and assembled chloroplast vectors were screened by PCR for the presence of CrAra h 1, CrAra h 1_171–586_ or CrAra h 2. (d) Western blot analysis of soluble protein extracts from parental and four isolates of transformed algae for CrAra h 1_171–586_ or CrAra h 2 with anti‐FLAG antibodies. JAG231—CrAra h 1. JAG234—CrAra h 1_171–586_. JAG194—CrAra h 2.

We reverse‐translated the peptide sequences of Ara h 1 and Ara h 2 from *Arachis hypogaea* using a *C. reinhardtii* chloroplast codon bias (see materials and methods). Codon optimization has been shown to increase transgene expression in algal chloroplasts (Franklin *et al*., 2002). Codon‐optimized *Ara h 1* and *Ara h 2*, hereafter referred to as *CrAra h 1* and *CrAra h 2*, respectively, and a truncated *CrAra h 1* consisting of amino acids 171–586 (*CrAra h 1*_*171‐586*_) encoding the core domain of Ara h 1 (Figure 1a) were separately cloned into chloroplast expression vector pJAG15 (Figure 1b (Gregory *et al*., 2012)) and confirmed by Sanger sequencing. This expression cassette adds a carboxy terminal TEV protease site followed by a FLAG‐tag and confers kanamycin resistance. Stable transgene integration into the chloroplast genome at the *psbA* locus is achieved via homologous recombination. Thus, transcription is controlled by the light dependent *psbA* promoter and 5′ and 3′ untranslated regions (UTRs; Figure 1b). Successful integration of CrAra h 1 (JAG231), CrAra h 1_*171–586*_ (JAG234) and CrAra h 2 (JAG194) into the plastid genome using particle bombardment was confirmed by PCR (Figure 1c). Four isolates of each transgenic algal strain were screened for recombinant protein accumulation by Western blot using anti‐FLAG antibodies (Figure 1d–e). *C. reinhardtii* that produce CrAra h 1_*171–586*_ and CrAra h 2 were successfully isolated, but we were unable to detect CrAra h 1 protein accumulation in any of the screened isolates (data not shown). Previous structural studies of recombinant Ara h 1 from *Escherichia coli* suggest that full‐length recombinant Ara h 1 is less stable than the core domain (Chruszcz *et al*., 2011). Thus, full‐length CrAra h 1 may also be unstable in algal chloroplasts. The apparent molecular weight of monomeric CrAra h 1 h1_*171–586*_ as observed by SDS‐PAGE is slightly larger than the predicted 50 kDa (Figure 1d, arrow). The major CrAra h 2 band migrates near the predicted 22 kDa (Figure 1e, arrow). A minor fraction of CrAra h 1 and CrAra h 2 appear to assemble into dimers and higher molecular weight complexes, respectively. No bands were observed in the untransformed parental *C. reinhardtii* strain, indicating successful production of these peanut allergens.

### Characterization of algal‐produced peanut allergens CrAra h 1 h1_*171–586*_ and CrAra h 2

**Figure 2:**
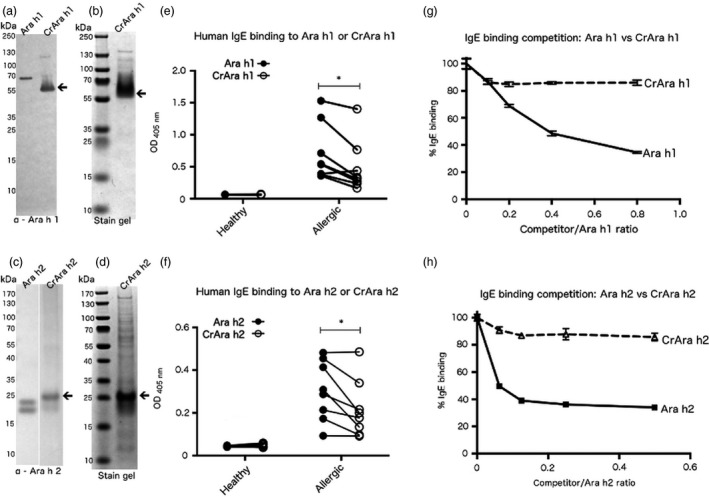
Immunoblot, Coomassie blue staining and IgE binding of purified CrAra h 1_171–586_ and CrAra h 2. FLAG affinity‐purified CrAra h 1_171–586_ (a–b) or CrAra h 2 (c–d) was separated by SDS‐PAGE, transferred to nitrocellulose and detected by Western blot using allergen‐specific antibodies or Coomassie blue staining. IgE that recognizes (e) Ara h 1 or CrAra h 1_171–586_, or (f) Ara h 2 or CrAra h 2 from serum in healthy controls or peanut‐allergic patients was detected by ELISA. Statistical significant was calculated using a paired *t*‐test. (g–h) Competition ELISA—equal volumes of serum from peanut‐allergic patients were pooled and pre‐incubated with increasing concentrations of (g) Ara h 1 or CrAra h 1_171–586_, or (h) Ara h 2 or CrAra h 2 and added to ELISA plates precoated with the corresponding native allergen. Each sample was tested in triplicate. *p = .05.

Affinity‐purified CrAra 1 h1_*171–586*_ (hereafter referred to as CrAra h 1‐core) and CrAra h 2 were analysed by Western blot using Ara h 1 or Ara h 2 specific antibodies, respectively, and compared to the native proteins. Ara h 1 and Ara h 2 were purified from peanuts as previously described (Hurlburt *et al*., 2014). Ara h 1 specific antibodies recognize both Ara h 1 and CrAra h 1‐core (Figure 2a); Ara h 1 migrates near the predicted 70 kDa and CrAra h 1‐core migrates slightly larger than the predicted 50 kDa (Figure 2a, arrow). Purified CrAra h 1‐core resolved by SDS‐PAGE and stained with Coomasie blue revealed a single major species (Figure 2b, arrow) and minor larger molecular weight species. Similarly, antibodies specific for Ara h 2 recognize both Ara h 2 and CrAra h 2. The 16‐kDa and 22‐kDa isoforms of Ara h 2 were detected by Western blot (Figure 2c). CrAra h 2 also appears as a doublet; the larger species migrates near the predicted 22 kDa for CrAra h 2 (Figure 2c, arrow), but the smaller CrAra h 2 species is likely a degradation product of full‐length CrAra h 2. Larger molecular weight species of CrAra h 2 observed in Western blots of algal lysates were less prevalent. SDS‐PAGE separation followed by Coomassie blue staining of affinity‐purified CrAra h 2 revealed the 22‐kDa CrAra h 2 as the major species (Figure 1d, arrow); however, multiple minor species were also observed.

CrAra h 1‐core and CrAra h 2 were evaluated for the presence of human IgE binding epitopes using serum from eight peanut‐allergic patients (hereafter referred to as allergic) obtained from the Food Allergy Resource Initiative repository (Table S1). Ara h 1‐ and Ara h 2‐specific IgE from patient samples had at least twofold higher OD than nonallergic controls as measured by ELISA (data not shown). IgE binding to native and algal‐produced allergens was observed in serum from allergic patients, but not controls (Figure 2e–f); IgE binding to CrAra h 1‐core and CrAra h 2 was significantly reduced compared to the native proteins. However, differences in sample purity could account for the disparate IgE binding. We therefore tested the relative affinity of IgE for naïve versus algal‐produced Ara h 1 and Ara h 2 more directly using a competition ELISA. Briefly, pooled serum was pre‐incubated with native or recombinant allergens at increasing concentrations and subsequently added to an ELISA plate precoated with Ara h 1 or Ara h 2. Increasing the concentration of Ara h 1 or Ara h 2 in pre‐incubated serum decreased IgE binding to plate‐bound allergen by approximately 60% at the highest concentrations tested (Figure 2g–h). CrAra h 1‐core and CrAra h 2 decreased binding to plate‐bound Ara h 1 or Ara h 2, respectively, by <20% at equivalent concentrations. These results suggest that IgE from allergic patients preferentially bind to native allergens over algal‐produced allergens.

### Immunotherapy using algal‐produced Ara h 1

**Figure 3:**
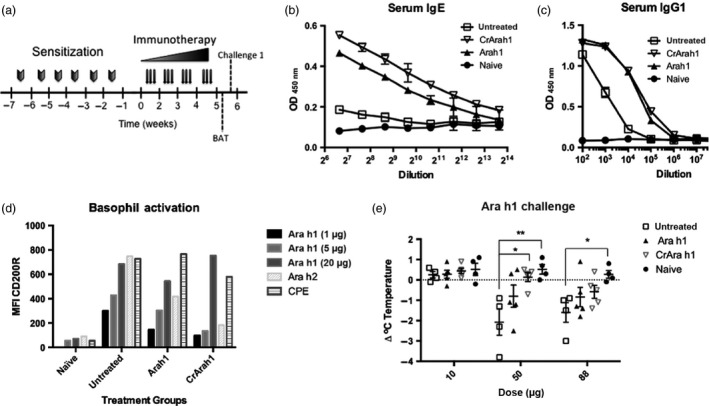
Impact of Ara h 1 or CrAra h 1_171–586_ immunotherapy on Ara h 1‐specific IgE, basophil activation and anaphylaxis in peanut sensitized mice. (a) Sensitization, immunotherapy, and Ara h 1 basophil activation and challenge schedule. Mice were sensitized to peanut using weekly exposures to peanut extract through the skin for 6 weeks followed by 4 weeks of immunotherapy—1 μg (weeks 1 and 2), 2 μg (week 3), 5 μg (week 4) of Ara h 1 or the molar equivalent of CrAra h 1 h1_171–586_. Blood was pooled from each group prior to challenge and tested for (b) Ara h 1‐specific serum IgE and (c) IgG1 by ELISA and (d) basophil activation by Ara h 1, Ara h 2 or peanut extract (CPE) as measured by an increase in CD200R by flow cytometry. Purified Ara h 1 was used in a dose escalation challenge (e). Anaphylaxis was measured as a drop in body temperature compared to baseline 30 min after each challenge 1 week after completing immunotherapy. Results are displayed as individual data points and average ± SEM are shown. Statistical significance was calculated using one‐way ANOVA followed by Bonferroni correction for multiple comparisons. *p < .05, **p < .01.

We employed a mouse model of peanut allergy to test whether CrAra h 1‐core could be used for desensitization to native Ara h 1. Similar to humans, peanut sensitized mice have increased peanut‐specific IgE, basophils that are activated by peanut allergens, and suffer anaphylaxis upon peanut exposure. Recent studies suggest that exposure to peanut through the skin may contribute to the initial sensitization. We therefore utilized a model of peanut allergy whereby mice are sensitized through weekly skin exposure (Tordesillas *et al*., 2014). This model does not require the use of adjuvants or tape stripping to induce skin damage and inflammation. Following sensitization, mice were treated with increasing doses of Ara h 1 or CrAra h 1‐core or left untreated for 4 weeks (Figure 3a; see Materials and methods). Prior to allergen challenge, blood was pooled from each group and tested for Ara h 1‐specific IgE and IgG1 and allergen‐specific basophil activation. In pooled samples, we observed slightly higher serum IgE levels in CrAra h 1‐core‐treated mice compared to Ara h 1‐treated mice, and both of these groups had elevated serum IgE compared to sensitized but untreated controls (Figure 3b). Ara h 1‐specific IgE was not detected in naïve mice. IgG1 levels were elevated in both native and CrAra h 1‐core‐treated groups compared to untreated controls (Figure 3c). Basophil activation was measured by flow cytometry as the median fluorescence intensity (MFI) of CD200R expression (Figure 3d). Briefly, we gated out B and T cells (CD19 and CD3, respectively) and selected IgE and CD49b positive cells. CD200R is expressed on murine basophils and is up‐regulated upon cross‐linking to allergen and subsequent activation. We observed increased Ara h 1‐dependent activation in basophils from sensitized compared to naïve mice, and activation was moderately reduced in Ara h 1‐ and CrAra h 1‐core‐treated mice, despite elevations in allergen‐specific IgE. We also observed a reduction in activation by Ara h 2 in basophils from Ara h 1‐ and CrAra h 1‐core‐treated mice, suggesting CrAra h 1‐core induces bystander suppression.

Systemic challenge using Ara h 1, Ara h 2 or peanut extract induces anaphylaxis in mice that are sensitized to peanut through the skin, which can be measured by a drop in core body temperature. We performed a dose escalation challenge using purified Ara h 1 one week post‐immunotherapy to test whether Ara h 1 or CrAra h 1‐core immunotherapy could protect peanut sensitized mice from anaphylaxis. Rectal temperatures were recorded at baseline and 30 min after each challenge (Figure 3e). Decreases in body temperature indicative of anaphylaxis were observed at the 50‐ and 88‐μg dose (highest dose possible due to the concentration of purified Ara h 1 used). Surprisingly, CrAra h 1‐core‐treated mice, but not Ara h 1‐treated mice, were significantly protected from anaphylaxis after a 50‐μg challenge. Statistical significance was lost at the highest dose for all but the naïve mice. Thus, CrAra h 1‐core immunotherapy protects peanut sensitized mice from Ara h 1‐induced anaphylaxis.

**Figure 4:**
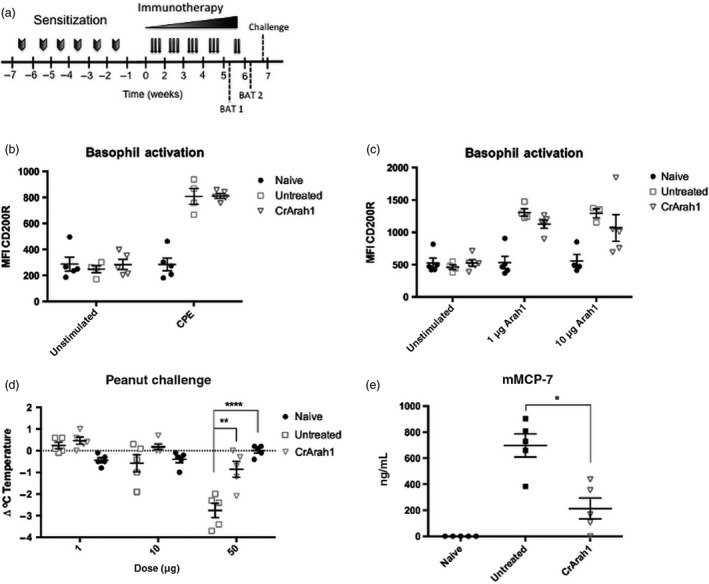
Impact of CrAra h 1 _171–586_ immunotherapy on basophil activation and anaphylaxis by peanut extract in peanut sensitized mice. (a) Sensitization, immunotherapy, basophil activation and challenge schedule. Sensitization and immunotherapy were performed as before except in week 5, two additional doses were given following the first basophil activation. (b–c) Blood was drawn from each mouse and tested individually for basophil activation by Ara h 1 (BAT 1) or CPE (BAT 2) as measured by an increase in CD200R expression using flow cytometry. (d) Peanut extract was used in a dose escalation challenge 1 week after completing immunotherapy. Anaphylaxis was measured as a drop in body temperature compared to baseline 30 min after each challenge. (e) Blood was drawn immediately after the final challenge and mMCP‐7 levels were measured in serum by ELISA. Statistical significance for challenge data was calculated using a one‐way ANOVA followed by Bonferroni multiple‐comparison correction. mMCP‐7 levels were compared using a Mann–Whitney test. *p < .05, **p < .01, ****p < .0001.

We sought to determine whether immunotherapy using CrAra h 1‐core also provided protection against exposure to peanut extract. Mice were sensitized as before, and after 4 weeks of CrAra h 1‐core immunotherapy (Figure 4a), basophils from each mouse were tested individually (rather than in pooled groups as in Figure 3) for reactivity to peanut extract. We did not observe a reduction in peanut extract dependent basophil activation (Figure 4b). IgE and IgG1 levels were similar to those in Figure 3 (data not shown). Immunotherapy was continued for an additional week and each mouse was retested for basophil reactivity to Ara h 1. Similar to pooled groups tested previously (Figure 3c), activation appeared to be reduced in CrAra h 1‐core‐treated mice at both concentrations of Ara h 1 tested (Figure 4c). However, the reduction in basophil activation did not reach statistical significance. We next performed a dose escalation challenge using peanut extract and recorded rectal temperatures at baseline and 30 min after each challenge. Untreated mice showed a decrease in body temperature after the 10‐μg dose, which continued to drop after the 50‐μg dose, indicative of severe anaphylaxis (Figure 4d). In contrast, CrAra h 1‐core‐treated mice were protected at the 10‐μg dose and only a slight drop in temperature was observed after the 50‐μg dose. Mouse mast cell protease 7 (mMCP‐7) levels were measured in blood serum immediately following challenge as an additional marker of anaphylaxis. We observed a significant reduction in CrAra h 1‐core‐treated mice compared to untreated controls (Figure 4e). Thus, immunotherapy using CrAra h 1‐core provides bystander suppression to additional peanut allergens.

## Discussion

In this study, we demonstrate that algal chloroplasts can produce two of the major peanut allergens: the core domain of Ara h 1 and full‐length Ara h 2. Compared to the native allergens, these recombinant algal‐produced allergens have reduced binding to IgE from peanut‐allergic patients. We tested the efficacy of immunotherapy using the algal‐produced Ara h 1 core domain in an adjuvant‐free murine model of peanut allergy that sensitizes mice through the skin, a route that mimics sensitization in some humans. In this model, we found that algal‐produced Ara h 1 core, but not native Ara h 1, significantly protects peanut sensitized mice from anaphylaxis induced by purified Ara h 1 or peanut extract, which demonstrates that systemic treatment with algal‐produced Ara h 1 confers antigen specific and bystander suppression to peanut allergens.

Food allergens from different sources (e.g. peanut, egg and recombinant) have various physical and enzymatic properties that contribute to IgE binding and allergenicity, including glycosylation, binding to pattern recognition receptors, or protease activity. The inability of algal‐produced peanut allergens to outcompete native allergens for IgE binding suggests key differences in structure. CrAra h 1‐core contains 9 of the 12 known linear Ara h 1 IgE binding epitopes (Burks *et al*., 1997) and CrAra h 2 contains all of the known linear Ara h 2 IgE binding epitopes (Stanley *et al*., 1997). These linear epitopes are identical to those found on the native proteins. Thus, algal‐produced allergens may have fewer or malformed conformational IgE epitopes. Studies of predominant IgE binding epitopes on Ara h 1 and Ara h 2 have focused primarily on linear epitopes using short synthetic peptides (Flinterman *et al*., 2008; Lin *et al*., 2012). However, analysis of IgE binding epitopes from humans and rats using phage display libraries identified dominant epitopes that are conformational and not linear (Bogh *et al*., 2012, 2014).

Different post‐translational modifications could also lead to reduced IgE binding. The observed molecular weight of CrAra h 1‐core domain (55 kDa) is slightly larger than the predicted one (50 kDa), possibly indicating post‐translational modifications, although a discrepancy between actual and observed molecular weight using SDS‐PAGE is not uncommon. Native Ara h 1 is glycosylated at two amino acid residues and undergoes spontaneous modifications through a Maillard reaction leading to advanced glycation end products (AGEs), especially when roasted (Hebling *et al*., 2013). *C. reinhardtii* chloroplasts lack the machinery for glycosylation and AGE modifications are not present on recombinant Ara h 1 from *E. coli* (Mueller *et al*., 2013), which also has reduced IgE binding compared to native Ara h 1 (Chruszcz *et al*., 2011). The absence of glycosylation and AGEs on CrAra h 1 could therefore lead to decreased IgE affinity. Acetylation has been reported for proteins produced or shuttled to the chloroplast (Lehtimaki *et al*., 2015). Interestingly, acetylation of Art v 1, the major mugwort pollen allergen, reduces allergenicity *in vivo* and *in vitro* (Perovic *et al*., 2009). Further structural characterization will be necessary to elucidate the relationship of reduced IgE binding and the structure of algal‐produced peanut allergens to determine whether this phenomenon is specific to Ara h 1 and Ara h 2 or a more general characteristic of chloroplast‐produced proteins.

Allergen‐specific immunotherapy can reduce clinical symptoms through suppression of effector mast cells and basophils (Matsuoka *et al*., 2013). Algal Ara h 1 core domain may mitigate peanut‐induced anaphylaxis through elevated Ara h 1‐specific IgG1 and reduced mast cell activation. Indeed, peanut‐induced anaphylaxis from IP challenge is primarily mast cell and IgE‐dependent (Sun *et al*., 2007). Allergen‐specific IgE levels were in fact elevated in treated mice compared to untreated mice, a paradox which has also been observed after allergen immunotherapy in allergic patients (Vickery *et al*., 2014). IgG antibodies have been proposed to play a protective role in food allergy (Burton *et al*., 2014) and correlate with clinical protection following immunotherapy (Vickery *et al*., 2013). Allergen‐specific IgG can act directly through sequestration of allergen (Kucuk *et al*., 2012) or by reducing activation of mast cells, which are key mediators of allergic responses (Voehringer, 2013) in peanut allergy (Reber *et al*., 2013). Allergen‐specific IgG promote internalization of IgE‐FcεRI complexes (Uermosi *et al*., 2014) and reduce downstream signalling through the low‐affinity Fcγ receptor IIB (Uermosi *et al*., 2010). Both Ara h 1‐ and CrAra h 1‐core‐treated mice had elevated levels of IgG1 compared to untreated controls. Thus, increases in Ara h 1‐specific IgG is not sufficient for protection in our model. Foxp3+ regulatory T cells have been shown to be suppressive in other models of allergen immunotherapy for food allergy (Mondoulet *et al*., 2015) and future studies should investigate their role in the context of algal‐produced allergens.

Our studies demonstrate the utility of algae as a host for producing allergens for peanut immunotherapy, but the limitations in this study should be considered. Administration of peanut allergens systemically for immunotherapy is not feasible in humans due to risk of adverse reactions (Nelson *et al*., 1997). However, the production of these allergens in algae allows for oral or sublingual delivery without extensive purification of allergens. Whole‐cell oral delivery using algal cells has been used to induce mucosal and systemic immune responses, demonstrating the bioavailability of proteins delivered by this approach (Dreesen *et al*., 2010; Gregory *et al*., 2013). Oral delivery of recombinant human blood proteins using tobacco induces tolerance through regulatory T cells (Wang *et al*., 2015). Systemic allergen challenge is also not a physiologic route of exposure, but we anticipate that interventions that can successfully suppress reactions to systemic allergen would be effective independent of route of exposure. An oral route of allergen challenge may be useful in future to address mucosal‐specific protective mechanisms such as peanut‐specific IgA that may be induced in the context of oral or sublingual immunotherapy.

## Experimental procedures

### Plasmid construction

Ara h 1 (accession: P43238) and Ara h 2 (accession: AAN77576) peptide sequences were reverse‐translated using Gene Designer (DNA 2.0 Inc., Menlo Park, CA) and synthesized (GeneWiz, La Jolla, CA). Codon optimization was accomplished using a reference set for the chloroplast of *C. reinhardtii* and verified by calculating the codon adaptation index (CAI (Puigbo *et al*., 2008); CrAra h 1: 0.93, CrAra h 2: 0.951). CrAra h 1 and CrAra h 2 were cloned into pJAG15 (Gregory *et al*., 2012) to yield pJAG231 and pJAG194, respectively, after digestion with NdeI and AgeI. The core domain of Ara h 1 (accession 3S7E_A) was amplified by PCR and cloned into pJAG15 to yield pJAG234. Correct assembly of all plasmids was verified by Sanger sequencing (GeneWiz). Descriptions of the completed chloroplast integration vectors can be found in Figure 1.

### *Chlamydomonas reinhardtii* growth and transformation

All strains were grown in Tris Acetate Phosphate (TAP) media at room temperature on a rotary shaker with or without light as indicated. *C. reinhardtii* strain *ΔpsbA* (Gregory *et al*., 2013) was used for all transformations, which were carried out by particle bombardment (Boynton *et al*., 1988). Briefly, 550‐nm or 1000‐nm gold particles were coated with the appropriate plasmid DNA as recommended by the manufacturer (Seashell Technologies, San Diego, CA) and shot into 5 × 10^7^ cells plated on TAP agar plates supplemented with 100 μg/mL kanamycin using gene gun (Bio‐Rad, Hercules, CA). Plates were grown in the dark for 24–48 h followed by constant illumination until colonies appeared (6–10 days). Colonies were patched onto TAP agar plates supplemented with 150 μg/mL kanamycin. Colonies that continued to grow were screened using gene‐specific primers as previously described (Rasala *et al*., 2010).

Large‐scale cultures were performed in 20‐l photobioreactors. Photobioreactors were constructed as previously described (Gregory *et al*., 2012). Circulation was provided using forced air (Gast Manufacturing, Benton Harbor, MI; Air pump model: DOA‐P704‐AA) through a 0.2‐μm filter. Cultures were grown to mid‐log phase and switched to constant illumination for 24–48 h before harvesting. Cells were harvested using a peristaltic pump (Cole‐Parmer, Vernon Hills, IL; Masterflex model 73700‐62) and the volume was reduced to ~ 600 mL using a continuous‐flow centrifuge (WVO Designs, North Charleston, SC; model Extreme Raw Power) at 6000 rpm. Cells were then pelleted by centrifugation and snap frozen using liquid nitrogen and stored at ‐80 °C.

### Western blotting and affinity purification

Transformed *C. reinhardtii* strains were screened for CrAra h 1 and CrAra h 2 protein accumulation by Western blot. Briefly, 10 mL of TAP media supplemented with 50 μg/mL kanamycin in test tubes was inoculated from plates and grown on a roller drum (Fischer Scientific, model 1640) for 48–72 h in the dark and switched to constant illumination for 24 h. Harvested cells were resuspended in lysis buffer (50 mm Tris pH 8.0, 400 mm NaCl, 0.5% Tween‐20, protease inhibitor cocktail (Sigma, St. Louis, MO) and lysed by sonication (Qsonica, Newtown, CT; Microson ultrasonic cell disruptor). Lysates were cleared by centrifugation, prepped with Novex LSD sample buffer (Life Technologies, Carlsbad, CA) supplemented with 5% β‐mercaptoethanol, heated to 85 °C for 10 min and resolved using NuPage Bis‐Tris 4–12% precast gels (Life Technologies). Samples were transferred to nitrocellulose, blocked with 5% milk in Tris‐buffered saline (50 mm Tris, 150 mm NaCl) with 0.1% Tween‐20 (TBS‐T) and probed with a mouse anti‐Flag mAb (Sigma) followed by alkaline phosphatase‐conjugated anti‐mouse IgG. Blots were visualized using nitroblue tetrazolium (NBT) and 5‐bromo‐4‐chloro‐3‐indolyl phosphate (BCIP) in alkaline phosphatase buffer.

CrAra h 1 and Cr Ara h 2 were purified from 20‐l photobioreactor cultures (described above) as follows. Frozen pellets were resuspended in lysis buffer with protease inhibitors and lysed by sonication (Branson Ultrasonics, Danbury, CT; model 450). M2 anti‐Flag affinity resin (Sigma) washed with lysis buffer was added to cleared lysates and rotated end over end at 4 °C for 2–4 h. Resin was pelleted by centrifugation and washed three times with 20 column volumes with lysis buffer and once with lysis buffer without Tween. Resin was collected by filtration in 5 mL polypropylene columns and eluted with 100 mm glycine pH 3.5 with 400 mm NaCl. Eluted fractions were neutralized with 1 m Tris pH 8.0 to a final concentration of 50 mm and tested for protein by Western blot. Fractions were combined and buffer exchanged to phosphate‐buffered saline (PBS) pH 7.4 using Vivaspin 6 columns (GE Healthcare Life Sciences, Pittsburgh, PA) with a 10‐kDa molecular weight cut‐off. Protein concentration was determined using the Bio‐Rad Protein DC assay and analysed by SDS‐PAGE followed by Western blot with allergen‐specific antibodies or stained with Imperial protein stain (Thermo Scientific, Somerset, NJ).

### Human IgE binding and competition assays

Immulon 4HBX ELISA plates (Thermo Scientific) were coated with purified CrAra h 1‐core (5 μg/mL), Ara h 1 (5 μg/mL), CrAra h 2 (1 μg/mL) or Ara h 2 (1 μg/mL) overnight at 4 °C. Plates were blocked with PBS containing 0.5% Tween and 1% BSA, washed with PBS‐T and then incubated overnight at 4 °C with serum from peanut‐allergic patients diluted in PBS at 1:5 or 1:10 for Ara h 1 or Ara h 2, respectively. IgE binding was detected with alkaline phosphatase‐conjugated anti‐human IgE (Sigma A3525) and visualized with para‐nitrophenyl phosphate (PNPP) at 1 mg/mL in PNPP substrate buffer (Invitrogen, Carlsbad, CA). Absorbances were measured at 405 nm using a Polarstar Omega spectrophotometer (BMG Labtech, Ortenberg, Germany). For competition assays, ELISA plates were coated with 5 μg/mL Ara h 1 or Ara h 2 as above. Equal volumes of serum from each patient were pooled and diluted 1:10. Pooled serum was incubated overnight at 4 °C with 0, 1, 2, 4 or 8 μg/mL of Ara h 1 or the molar equivalent of CrAra h 1‐core. For Ara h 2, pooled serum was incubated overnight at 4 °C with 0, 0.625, 1.25, 2.5 or 5 μg/mL or the molar equivalent of CrAra h 2. Allergen/pooled serum mixes were added to Ara h 1 or Ara h 2 coated plates in triplicate and incubated overnight at 4 °C. Plates were washed and IgE binding was detected as described above.

### Mouse peanut sensitization and immunotherapy

All animal procedures were approved by the Institutional Animal Care and Use Committee of the Icahn School of Medicine at Mount Sinai under protocol 13‐1546. Six‐week‐old female C3H/HeJ mice were ordered from the National Cancer Institute. Sensitization was carried out as previously described (Tordesillas *et al*., 2014). The sensitization and immunotherapy schedule is outlined in Figure 3a and 4a. Briefly, mice were anesthetized, abdominal fur was removed with depilatory cream, and 500 μg of peanut extract was applied in PBS and allowed to dry. Purified native or algal‐produced allergens were diluted in PBS and administered by intraperitoneal injection three times weekly as follows: 1 μg Ara h 1 (or molar equivalent of CrAra h 1‐core)/injection during week 1–2, 2 μg/injection in week 3, and 5 μg/injection thereafter.

### Basophil activation

Basophil activation was performed as previously described (Leonard *et al*., 2012). Briefly, blood was drawn from each mouse and tested individually or pooled as indicated. Samples were diluted with RPMI and incubated with purified allergens or extract at the indicated concentrations for 90 min at 37 °C. Activation was stopped with PBS containing EDTA. Red blood cells were lysed using IMMUNO‐LYSE (Beckman Coulter, Jersey City, NJ) as indicated by the manufacturer. Cells were washed and Fc receptors blocked with anti‐CD16/CD32. T and B cells were gated out with anti‐CD3‐APC‐Cy7 and anti‐CD19‐APC‐Cy7 and basophils were selected using anti‐IgE‐FITC (clone 23G3) and anti‐CD49b‐APC (clone DX5). CD200R‐PE (clone ox‐110) was used as a maker for basophil activation. Antibodies were purchased from eBioscience (San Diego, CA).

### Quantification of immunoglobulin titres

Immulon 4HBX ELISA plates were coated with 1 μg/mL purified native Ara h 1 overnight at 4 °C and blocked with PBS‐T with 1% BSA. Pooled serum was tested in triplicate at a 1:100 dilution and each subsequent twofold dilution until 1:12,800. IgE binding was detected with rat anti‐mouse IgE (BD Pharmingen, San Jose, CA; clone R35‐72) followed by horse radish peroxidase (HRP)‐conjugated anti‐rat IgG. IgG1 binding was detected with biotin‐conjugated anti‐mouse IgG1 followed by streptavidin‐conjugated‐HRP. Binding was visualized using 1‐step TMB substrate (eBioscience) and absorbances were measured at 450 nm using a Polarstar Omega spectrophotometer (BMG Labtech).

### Allergen challenge and assessment of anaphylaxis

Mice were systemically challenged via intraperitoneal injection using 10‐, 50‐ and 88‐μg doses of purified native Ara h 1 diluted in PBS (at the prepared concentration, 88 μg was the maximum dose that could be administered using 200 μL). Body temperature was measured using a rectal thermometer (WPI Instruments, Sarasota, FL; model BAT‐12) prior to challenge and thirty minutes after each challenge was administered. A drop in body temperature is indicative of systemic anaphylaxis.

### Quantification of mMCP‐7

ELISA plates were coated overnight at 4 °C with anti‐mouse tryptase β1/MCPT7 (R&D Systems AF1937) and blocked with PBS‐T with 1% BSA. Serum was diluted in PBS, added to the coated plate and incubated overnight at 4 °C. Recombinant mMCP‐7 (R&D Systems 1937‐SE‐20) was used as a standard. mMCP‐7 was detected with biotin‐conjugated anti‐mouse tryptase β1/MCPT7 (R&D Systems, Minneapolis, MN; BAF1937) followed by HRP‐conjugated avidin and visualized using 1‐Step TMB substrate. Absorbances were measured at 450 nm using a Polarstar Omega spectrophotometer (BMG Labtech).

### Statistics

Statistical analyses were calculated using GraphPad Prism software. Human IgE binding was compared using a paired t‐test. Statistical significance for allergen challenge was calculated using one‐way ANOVA followed by Bonferroni correction for multiple comparisons. mMCP‐7 levels were compared using a Mann–Whitney test.

## Supporting information


**Table S1** Food Allergy Resource Initiative Samples. Peanut allergic.
